# Triglyceride Glucose-Body Mass Index Is a Simple and Clinically Useful Surrogate Marker for Insulin Resistance in Nondiabetic Individuals

**DOI:** 10.1371/journal.pone.0149731

**Published:** 2016-03-01

**Authors:** Leay-Kiaw Er, Semon Wu, Hsin-Hua Chou, Lung-An Hsu, Ming-Sheng Teng, Yu-Chen Sun, Yu-Lin Ko

**Affiliations:** 1 The Division of Endocrinology and Metabolism, Department of Internal Medicine, Taipei Tzu Chi Hospital, Buddhist Tzu Chi Medical Foundation, New Taipei City, Taiwan; 2 Department of Research, Taipei Tzu Chi Hospital, Buddhist Tzu Chi Medical Foundation, New Taipei City, Taiwan; 3 Department of Life Science, Chinese Culture University, Taipei, Taiwan; 4 The Division of Cardiology, Department of Internal Medicine and Cardiovascular Center, Taipei Tzu Chi Hospital, Buddhist Tzu Chi Medical Foundation, New Taipei City, Taiwan; 5 The First Cardiovascular Division, Department of Internal Medicine, Chang Gung Memorial Hospital and Chang Gung University College of Medicine, Taoyuan, Taiwan; 6 Department of Laboratory Medicine, Chang Gung Memorial Hospital, Taipei, Taiwan; 7 School of Medicine, Tzu Chi University, Hualien, Taiwan; University of Catanzaro Magna Graecia, ITALY

## Abstract

**Background:**

Insulin resistance (IR) and the consequences of compensatory hyperinsulinemia are pathogenic factors for a set of metabolic abnormalities, which contribute to the development of diabetes mellitus and cardiovascular diseases. We compared traditional lipid levels and ratios and combined them with fasting plasma glucose (FPG) levels or adiposity status for determining their efficiency as independent risk factors for IR.

**Methods:**

We enrolled 511 Taiwanese individuals for the analysis. The clinical usefulness of various parameters—such as traditional lipid levels and ratios; visceral adiposity indicators, visceral adiposity index (VAI), and lipid accumulation product (LAP); the product of triglyceride (TG) and FPG (the TyG index); TyG with adiposity status (TyG-body mass index [BMI]) and TyG-waist circumference index [WC]); and adipokine levels and ratios—was analyzed to identify IR.

**Results:**

For all lipid ratios, the TG/high-density lipoprotein cholesterol (HDL-C) ratio had the highest additional percentage of variation in the homeostasis model assessment of insulin resistance (HOMA-IR; 7.0% in total); for all variables of interest, TyG-BMI and leptin-adiponectin ratio (LAR) were strongly associated with HOMA-IR, with 16.6% and 23.2% of variability, respectively. A logistic regression analysis revealed similar patterns. A receiver operating characteristic (ROC) curve analysis indicated that TG/HDL-C was a more efficient IR discriminator than other lipid variables or ratios. The area under the ROC curve (AUC) for VAI (0.734) and TyG (0.708) was larger than that for TG/HDL-C (0.707). TyG-BMI and LAR had the largest AUC (0.801 and 0.801, respectively).

**Conclusion:**

TyG-BMI is a simple, powerful, and clinically useful surrogate marker for early identification of IR.

## Introduction

Insulin resistance (IR) involves reduced muscle and adipose tissue sensitivities to insulin and reduced ability of the liver to suppress hepatic glucose production and output [1]. IR is considered a major risk factor for type 2 diabetes and cardiovascular (CV) diseases [2]. Because of the clinical importance of IR, the ability to identify individuals with IR before the development of cardiometabolic diseases is of paramount importance. Although the hyperinsulinemic euglycemia clamp remains the gold standard for measuring IR [3], its practical clinical application is limited by the labor intensiveness and cost and by ethical concerns. Therefore, a simple, reliable, and reproducible index for measuring IR is urgently required.

It has been demonstrated that the product of TG and FPG levels (TyG index) presents moderate power as a surrogate marker for estimating HOMA-IR index in healthy subjects [4]. And The TyG index has high sensitivity and specificity compared with the euglycemic hyperinsulinemic clamp test for recognizing IR [5]. The superiority of the TyG index in identifying IR was also reported in many other studies [6,7,8]. With these findings, a highly efficient substitute measure to establish IR can be easily applied in clinical setting.

Previous studies also proposed other new formulas for estimating IR from adiposity. The visceral adiposity index (VAI), a mathematical model used to identify IR, is based on the anthropometric body mass index (BMI), waist circumference (WC), and metabolic (TG and HDL-C) parameters [9,10]. The lipid accumulation product (LAP), another mathematical model calculated from the WC and fasting TG levels, has been demonstrated as an alternative index for lipid accumulation and as a surrogate marker for IR [11,12]. Recently, a cross-sectional study directly compared lipid ratios, VAI, LAP, and TyG for measuring their IR detection efficiency, and the TyG was revealed to be the most efficient marker for early identification of IR [6]. BMI and WC are simple, inexpensive and noninvasive anthropometric parameters and are commonly adopted as useful indicators of obesity and other metabolic risk. As evidence increasingly suggests that obesity exhibits a close relationship with IR and because TyG are well-established as promising surrogate marker of IR, a combination of obesity and TyG can potentially identify IR more strongly than other surrogate markers. Thus, in this investigation, we compare lipid, adipokines, and lipid and adipokine ratios, visceral adiposity indicators, TyG and TyG related parameters (TyG-WC, TyG BMI) for early identification of IR. We analyzed whether a combination of TyG with BMI or WC can be a more powerful, simple and inexpensive clinical surrogate marker for IR.

## Subjects and Methods

### Study population

This study was approved by the institutional review board of the Taipei Tzu Chi Hospital, Buddhist Tzu Chi Medical Foundation (IRB number: 98-IRB-021-XD). Participants were recruited during cardiovascular health examinations between October 2003 and September 2005 at Chang Gung Memorial Hospital. All the study individuals provided written informed consent. Medical history- and lifestyle characteristics-related questionnaire responses were collected. Cancer; current renal or liver disease; and a history of myocardial infarction, stroke, or transient ischemic attacks were the exclusion criteria. An initial recruitment revealed 617 individuals. In addition, participants with diabetes mellitus (defined as fasting blood glucose≧7.0 mmol/L (7 individuals) or the regular use of medications for diabetes mellitus (11 individuals)), aged <18 years (5 individuals), triglyceride levels > 500 mg/dL (10 individuals) and a history of regular medication for hypertension (66 individuals) or hyperlipidemia (9 individuals) were excluded. Finally, we enrolled 511 people—257 men and 254 women—for the analysis. Blood pressure was measured with patients in the sitting position after a 15-min rest period. Weight was measured on a calibrated beam scale with participants wearing light clothing, and height was measured without shoes. BMI was calculated by dividing weight (in kilograms) by the square of height (in meters). WC was measured using an inelastic tape at the midpoint of the bottom of the rib cage and the top of the iliac crest. Fasting blood samples were obtained from each participant. Current smoker was defined as smoking at least 1 cigarette per day at the time of survey. Alcohol consumption was defined as anyone who consumed alcohol once a day or more. Exercise was defined as regular exercise one or more times per week, at least 20 min for each time. Table 1 summarizes the clinical and biometric features of the study group stratified by sex and IR.

**Table 1:** Baseline Characteristics of the Study Subjects.

	Men	Women	*P*	Non-IR	IR	*P*
Number	257	254		405	106	
Age, year	43.0 (38.1–50.0)	46.0 (40.0–51.2)	0.002	45.0 (39.0–51.0)	44.7 (39.8–50.0)	0.974
Body mass index, kg/m2	24.8 (22.8–26.4)	22.8 (20.8–25.5)	< 0.001	23.3(21.3–25.4)	26.5(24.5–28.7)	< 0.001
Waist circumference, cm	87.0 (82.0–91.0)	80.0 (74.0–87.3)	< 0.001	83.0 (76.0–88.5)	90.0 (84.8–97.3)	< 0.001
Systolic BP, mmHg	112.0 (102.0–122.0)	110.0 (98.0–122.0)	0.077	108.0 (100.0–122.0)	116.0 (108.0–130.0)	< 0.001
Diastolic BP, mmHg	76.0 (70.0–84.0)	72.0 (66.0–80.0)	< 0.001	72.0 (66.0–82.0)	80.0 (72.0–86.0)	< 0.001
Mean BP, mmHg	88.7 (81.0–95.3)	84.7 (77.2–94.2)	0.002	85.3 (78.0–93.3)	92.3 (85.7–98.7)	< 0.001
TC, mg/dL	200.0 (176.0–221.0)	192.0 (170.0–219.5)	0.085	195.0 (172.0–220.0)	198.0 (178.5–223.0)	0.319
HDL-C, mg/dL	49.0 (42.5–55.0)	62.0 (52.0–71.0)	< 0.001	57.0 (47.0–68.0)	47.0 (39.0–56.0)	< 0.001
LDL-C, mg/dL	120.0 (98.0–140.0)	109.0 (91.8–134.3)	0.004	114.0 (93.5–137.0)	119.0 (95.0–139.0)	0.417
TG, mg/dL	131.0 (91.0–193.5)	87.0 (65.0–129.3)	< 0.001	98.0 (68.5–150.0)	140.0 (101.5–220.5)	< 0.001
Non-HDL-C, mg/dL	153.0 (124.0–171.5)	131.5 (109.0–155.0)	< 0.001	137.0 (114.0–163.0)	153.0 (130.8–175.3)	0.001
TC/HDL-C	4.13 (3.39–4.89)	3.12 (2.69–3.78)	< 0.001	3.40 (2.83–4.18)	4.34 (3.48–4.94)	< 0.001
TG/HDL-C	2.78 (1.63–4.32)	1.43 (0.96–2.32)	< 0.001	1.69 (1.11–2.95)	3.29 (1.75–4.83)	< 0.001
LDL-C/HDL-C	2.50 (1.94–3.04)	1.78 (1.43–2.32)	< 0.001	2.00 (1.55–2.60)	2.53 (1.95–2.98)	< 0.001
Non-HDL-C/HDL-C	3.13 (2.39–3.89)	2.12 (1.69–2.78)	< 0.001	2.40 (1.83–3.18)	3.34 (2.48–3.94)	< 0.001
Current smokers, %	34.2	3.9	< 0.001	16.8	28.3	0.007
Alcohol consumption, %	5.1	0.4	0.001	2.7	2.8	0.949
Exercise %	31.9	31.9	0.973	32.3	30.2	0.085
Fasting plasma glucose, mg/dl	94.0 (89.0–99.0)	90.0 (85.0–95.0)	< 0.001	91.0 (87.0–95.0)	96.5 (92.0–105.0)	< 0.001
Fasting serum insulin, μU/mL	8.06 (6.29–10.94)	7.44 (5.72–10.27)	0.025	7.06 (5.56–8.56)	13.89 (12.18–18.00)	< 0.001
HOMA-IR index	1.89 (1.46–2.54)	1.69 (1.26–2.35)	0.002	1.58 (1.24–1.98)	3.33 (2.85–4.30)	< 0.001
Adiponectin, μg/mL	4.65 (3.13–7.02)	8.09 (5.60–11.55)	< 0.001	6.81 (4.44–10.05)	4.11 (2.67–6.28)	< 0.001
Leptin, ng/mL	9.17 (5.05–14.13)	24.40 (15.73–32.41)	< 0.001	13.19 (6.88–23.42)	21.87 (12.77–34.89)	< 0.001
Resisin, ng/mL	14.8 (10.2–22.4)	15.1 (10.7–22.6)	0.840	14.9 (10.4–22.7)	15.2 (10.0–21.6)	0.682
LAR	2.07 (0.92–3.67)	3.03 (1.57–5.29)	< 0.001	2.01 (0.98–3.49)	4.74 (3.27–9.24)	< 0.001
AR index	1.50 (1.31–1.74)	1.31 (1.03–1.53)	< 0.001	1.36 (1.09–1.62)	1.53 (1.36–1.80)	< 0.001
CRP, mg/L	0.64 (0.29–1.31)	0.50 (0.23–1.16)	0.050	0.47 (0.22–0.98)	1.19 (0.56–2.31)	< 0.001

IR, insulin resistance; BP, blood pressure; HDL-C, high-density lipoprotein cholesterol; LDL-C, low-density lipoprotein cholesterol; TC, total cholesterol; TG, triglyceride; HOMA-IR, homeostasis model assessment of insulin resistance; LAR, leptin-adiponectin ratio; AR index, adiponectin-resistin index; CRP, C-reactive protein. Data are presented as median (interquartile range), or percent.

### Laboratory examinations and assays

Lipid variables were performed according to the methods described by Teng et al. [13]. Non-HDL-C was calculated by subtracting high density lipoprotein cholesterol (HDL-C) from total cholesterol (TC). FPG was enzymatically determined by using the hexokinase method. Serum insulin levels were measured using an immunoradiometric assay (Bio-source, Nivelles, Belgium). Homeostasis model assessment of insulin resistance (HOMA-IR) was calculated using the following formula: HOMA-IR = fasting insulin (μU/mL) × FPG (mmol/L)/22.5 [14]. Insulin resistance was recognized when the HOMA-IR index reached the upper quartile. The C-reactive protein (CRP) level was measured by using a sandwich-type enzyme-linked immunosorbent assay (ELISA), which was developed in house. [15]. Serum leptin and adiponectin levels also were measured using an in-house sandwich-type ELISA assay. All in-house kits showed good correlation when compared with commercially available ELISA kits [15,16,17]. Circulating resistin levels were measured using commercially available ELISA kits (R&D Systems, Minneapolis, MN, USA). The within-day precision and day-to-day precision were 2.2% and 2.1% for TC, 3.4% and 4.5% for HDL-C, 2.3% and 3.0% for LDL-C, 2.0% and 2.2% for TG, 1.3% and 2.0% for glucose, 6.0% and 10.0% for insulin, 5.1% and 4.0% for leptin, 3.2% and 8.6% for resistin, 7.7% and 7.0% for adiponectin and 8.2% and 9.5% for CRP, respectively.

Visceral adiposity indicators, TyG related parameters and adipokine ratios were calculated as follows: LAP for men: (WC [cm]– 65) × TG [mmol/L]); LAP for women: (WC [cm]– 58) × TG [mmol/L]) [18]. VAI for men: (WC [cm]/39.68 + (1.88 × BMI) × (TG [mmol/L]/1.03) × (1.31/HDL-C [mmol/L]); VAI for women: (WC [cm]/36.58 + (1.89 × BMI) × (TG [mmol/L]/0.81) × (1.52/HDL-C [mmol/L]) [9]. TyG index: Ln [TG (mg/dL) × FPG (mg/dL)/2] [4,5]. TyG-BMI: TyG index × BMI. TyG-WC: TyG index × WC. LAR: leptin (ng/ml)/adiponectin (mg/ml) [19]. AR index: 1 + log10 (fasting resistin [ng/mL]) − log10 (fasting adiponectin [μg/mL]) [20].

### Statistical analyses

All statistical analyses were conducted using SPSS (Version 12.0 for Windows; SPSS, Chicago, IL, USA). Before conducting linear regression, all variables except TyG, TyG-BMI, and TyG-WC were log-transformed (natural logarithm) to adhere to a normality assumption. Multiple linear regression analyses with HOMA-IR as the dependent variable were performed to evaluate associations of these variables with HOMA-IR. The predictive values of each variable were judged by comparing the proportion of the total variation for each index; in other words, the R^2^ for a base model that excluded each of the indexes subtracted from the R^2^ for the entire regression model. We divided each index into increasing quartile values and calculated odds ratios (ORs) and 95% confidence intervals (CIs) for IR, and compared indexes in quartiles 2–4 with those in quartile 1 using logistic regression analysis. To detect individuals with elevated IR, we compared the relative diagnostic strengths of lipid parameters, lipid ratios, visceral adiposity indicators, TyG related parameters and adipokines by plotting receiver operating characteristic (ROC) curves. ROC curves were constructed and the area under the curves (AUCs) was calculated for each evaluated variable using a HOMA-IR index 2.43 as the reference value to define IR. The AUCs for all variables of interest were compared nonparametrically. The significant difference among AUCs was calculated using an online tool [http://vassarstats.net/roc_comp.html] [21]. A two-tailed P value < 0.05 was considered statistically significant.

## Results

BMI, WC, systolic, diastolic and mean BP, TG, non-HDL-C, TC/HDL-C, TG/HDL-C, low-density lipoprotein cholesterol (LDL-C)/HDL-C, non-HDL-C/HDL-C, current smokers, FPG, insulin, HOMA-IR, leptin, LAR, AR index and CRP were significantly higher, whereas HDL-C, and adiponectin were lower in insulin-resistant individuals than in their noninsulin-resistant counterparts (Table 1). BMI, WC, diastolic, and mean BP, TG, LDL-C, non-HDL-C, TC/HDL-C, TG/HDL-C, LDL-C/HDL-C, non-HDL-C/HDL-C, current smokers, alcohol consumption, FPG, insulin, HOMA-IR, AR index and CRP were significantly higher, whereas age, HDL-C, adiponectin, leptin, and LAR were lower in men than in women (Table 1).

After controlling for potential intermediate variables of lipid traits, studied parameters individually significantly predicted HOMA-IR except TC, LDL-C, and non-HDL-C (Table 2). The models with lipid ratios were consistently superior to those with single variables for predicting HOMA-IR. For all lipid ratios, such as TC/HDL-C, TG/HDL-C, LDL-C/HDLC, and non-HDL-C/HDL-C, the additional percentage of variation ranged from 0.8% to 7.0% in HOMA-IR, with TG/HDL-C being the strongest predictor. For visceral adiposity indicators and TyG related parameters, TyG-BMI had the strongest association with HOMA-IR, explaining 16.6% of the variability in HOMA-IR, followed by TyG-WC (14.2%), LAP (11.3%), VAI (9.7%) and TyG (8.5%). For adipokines, LAR had the strongest association with HOMA-IR, which explained 23.2% of the variability in HOMA-IR, followed by leptin (22.2%), adiponectin (6.6%), and the AR index (0.7%), whereas resistin was not associated with HOMA-IR.

**Table 2:** Multiple Linear Regression Models for Predicting Homeostatic Model Assessment.

	Additional R^2^	β	*P*
Lipid measures			
Ln TC	0.001	-0.199	0.434
Ln TG	0.059	0.476	< 0.001
Ln LDL-C	0.003	-0.041	0.170
Ln non-HDL-C	0.002	0.034	0.330
Ln HDL-C	0.043	-0.189	< 0.001
Lipid ratios			
Ln TC/HDL-C	0.024	0.157	< 0.001
Ln TG/HDL-C	0.070	0.087	< 0.001
Ln LDL-C/HDL-C	0.008	0.052	0.034
Ln non-HDL-C/HDL-C	0.026	0.094	< 0.001
Visceral adiposity indicators			
Ln LAP	0.113	0.096	< 0.001
Ln VAI	0.097	0.096	< 0.001
TyG related parameters			
TyG index	0.085	0.116	< 0.001
TyG-BMI	0.166	0.003	< 0.001
TyG-WC	0.142	0.001	< 0.001
Adipokines			
Ln Leptin	0.222	0.138	< 0.001
Ln Adiponectin	0.066	-0.092	< 0.001
Ln Resistin	-0.012	-0.013	0.370
Ln LAR	0.232	0.100	< 0.001
AR index	0.007	0.077	0.001

*P* value was adjusted for age, sex, smoking, systolic blood pressure, diastolic blood pressure, alcohol consumption, exercise and C-reactive protein.

TC, total cholesterol; TG, triglycerides; LDL-C, low-density lipoprotein cholesterol; HDL-C, high-density lipoprotein cholesterol; LAP, Lipid accumulation product; VAI, visceral adiposity index; TyG index, the product of triglycerides and fasting glucose; BMI, body mass index; WC, Waist circumference; LAR: leptin-adiponectin ratio; AR index: adiponectin-resistin index.

The direct comparative ORs and 95% CIs for those in the top quartiles of each variable are presented in Table 3. After adjusting for age, sex, smoking status, systolic and diastolic BP, alcohol consumption, exercise and CRP levels, each of the lipid traits or lipid ratios, including TG, HDL-C and TG/HDL-C were strongly associated with IR (*P* < 0.001), whereas TC, LDL-C, non-HDL-C, and LDL-C/HDL-C were not associated with IR. Among all lipid parameters, in terms of their strengths of association with IR, TG related parameters were more efficient overall than other lipid parameters, with TG and TG/HDL-C had the strongest association (top *vs*. bottom OR [95% CI]: 3.11 [1.58–6.11] and 2.98 [1.49–5.96], respectively). All visceral adiposity indicators and TyG related parameters were strongly associated with IR (*P* < 0.001) and TyG-BMI had the highest OR [95% CI] (6.10[2.51–14.80]). For adipokines, leptin, adiponectin, LAR, and the AR index were associated with IR, and LAR had the highest OR [95% CI] (12.99[3.95–42.70]).

**Table 3:** Adjusted Odds Ratios of Insulin Resistance among Those in the Lowest Quartile and Upper Three Quartiles of Each Variable.

	Odds ratio
Lipid measures	
TC	1.17 (0.68–2.02)
TG	3.11 (1.58–6.11)*
LDL-C	1.04 (0.60–1.79)
Non-HDL-C	1.59 (0.88–2.86)
HDL-C	0.26(0.15–0.44)*
Lipid ratios	
TC/HDL-C	2.23 (1.16–4.30)
TG/HDL-C	2.98 (1.49–5.96)*
LDL-C/HDL-C	1.83 (0.99–3.38)
non-HDL-C/HDL-C	2.23 (1.16–4.30)
Visceral adiposity indicators	
LAP	3.19 (1.56–6.55)
VAI	3.20 (1.57–6.55)*
TyG related parameters	
TyG index	4.11 (2.00–8.46)*
TyG-BMI	6.10 (2.51–14.80)*
TyG-WC	3.55 (1.71–7.36)*
Adipokines	
Leptin	5.60 (2.54–12.37)*
Adiponectin	0.32 (0.19–0.54)*
Resistin	0.73 (0.43–1.24)
LAR	12.99 (3.95–42.70)*
AR index	2.37 (1.31–4.29)

*P < 0.001

Abbreviations as in Table 2

*P* value was adjusted for age, sex, smoking, systolic blood pressure, diastolic blood pressure, alcohol consumption, exercise and C-reactive protein.

Data are presented as Odds ratio (95% confidence interval).

To detect each evaluated variables with IR, the ROC curves of the lipid parameters, lipid ratios, visceral adiposity indicators, TyG related parameters and adipokines were plotted, and the AUCs were compared (Table 4). The AUCs derived from the lipid ratios were in general significantly larger than those from individual lipids. For the lipid ratios, the AUC was the largest for TG/HDL-C (0.707). For all variables of interest, TyG-BMI and LAR had the largest AUC (0.801), followed by TyG-WC (0.772), LAP (0.761), VAI (0.743), TyG (0.708), and leptin (0.690). The TyG-BMI AUC in detecting IR was significantly higher than that of TyG, TG/HDL-C, and leptin (*P* = 0.004, 0.004 and < 0.001, respectively). The AUC of LAR in detecting IR was also larger than that of TyG, TG/HDL-C, and leptin (*P* = 0.003, 0.003 and < 0.001, respectively).

**Table 4:** Areas Under the Receiver Operating Characteristic (ROC) Curves for Each Evaluated Variable in Predicting Homeostatic Model Assessment.

	Area under the ROC curve
Lipid measures	
Ln TC	0.531**##
Ln TG	0.690**##
Ln LDL-C	0.526**##
Ln non-HDL-C	0.604**##
Ln HDL-C	0.313**##
Lipid ratios	
Ln TC/HDL-C	0.679**##
Ln TG/HDL-C	0.707*#
Ln LDL-C/HDL-C	0.645**##
Ln non-HDL-C/HDL-C	0.679**##
Visceral adiposity indicators	
Ln LAP	0.761
Ln VAI	0.734
TyG related parameters	
TyG index	0.708*#
TyG-BMI	0.801
TyG-WC	0.772
Adipokines	
Ln Leptin	0.690**##
Ln Adiponectin	0.293**##
Ln Resistin	0.487**##
Ln LAR	0.801
AR index	0.645**##

*P < 0.005 compare to TyG-BMI,

**P < 0.001 compare to TyG-BMI

^#^ *P <* 0.005 compare to Ln LAR,

^##^ *P <* 0.001 compare to Ln LAR

Abbreviations as in Table 2.

## Discussion

In this study, we compared lipids, adipokines, lipid and adipokine ratios, VAI, LAP, TyG, TyG-BMI, and TyG-WC as variables in identifying IR. Our investigation revealed that TyG-BMI, with the combination of TG, FPG, and adiposity status, was a more efficient surrogate marker for early identification of IR in nondiabetic Taiwanese people. Although LAR has a comparable effect on IR, it is clinically less useful because it is not routinely measured in clinical practice. Thus, our data showed that TyG-BMI is a simple and clinically useful surrogate marker for IR in nondiabetic individuals. In addition, the ROC curve investigation affirmed that TyG-BMI is the most favorable surrogate marker of IR.

Previous studies proposed that traditional lipid ratios, particularly the TG/HDL-C ratio based on the proportion between proatheroagenic and antiatherogenic fractions, are more effective than single lipids measures in identifying IR [6,22,23]. Concordant with these studies, our investigation revealed that the magnitude of the association was higher for TG related parameters, especially TG/HDL-C, than for any other lipid measures or ratios. Therefore, TG/HDL-C can be reasonably proposed as a simple and convenient IR surrogate marker. The mechanism through which hypertriglyceridemia and low HDL-C cause IR remains unresolved. One previous study has proposed that hypertriglyceridemia might cause fatty acid accumulation in non-adipose tissues such as the liver, muscle, and heart which resulted in ectopic lipid deposition with lipotoxicity that has been accepted as a mechanism for IR [24].

Adipose tissue has been recognized as a principal endocrine organ. By secreting an increasing number of adipokines, the adipose tissue is involved in the regulation of whole-body energy balance and insulin action [25]. The altered adipokine profile in obesity leads to profound changes in insulin sensitivity and various metabolic derangements. Leptin and adiponectin are the two most easily characterized adipokines that respond reciprocally to increasing adiposity [26]. Accumulating evidence suggests that hyperleptinemia and hypoadiponectinemia are associated with increased adiposity and IR in nondiabetic subjects [19]. The strength of associations between LAR and HOMA-IR is greater than that of leptin or adiponectin alone [19]. The AR index, combined with adiponectin and resistin levels, is a cost-effective and a precise diagnostic biomarker for insulin sensitivity and is a predictor of acute coronary syndrome [20]. Our data revealed that the LAR, not the AR index, is an extremely powerful indicator for IR; however, as leptin and adiponectin assays are more expensive than glucose and triglycerides assays, their clinical utility is limited.

Both VAI and LAP are visceral adiposity indicators involving lipid variables and adiposity status for identifying IR. VAI is a sex-specific index in which the BMI and WC measurements are combined with TG and HDL-C to identify IR and cardiometabolic risk [9,10]. LAP, calculated using WC and fasting TG levels, is an index for excessive lipid accumulation and is a favorable surrogate for recognizing IR in non-diabetic people [27] and for identifying diabetes or CV risk in individuals in previous studies [12,18]. Our research showed that VAI and LAP are more efficient than TG/HDL-C in recognizing IR because both parameters reflects abdominal obesity, particularly visceral fat, which is strongly correlated with IR.

Previous studies revealed that both TG and FPG levels are well validated for their roles in identifying IR [28,29]. High FPG is an independent risk factor for type 2 diabetes in people with normoglycemic levels [30]. In obese youth with normal FPG levels, insulin sensitivity declines when moving from low to high FPG [31]. With the combination of TG and FPG levels, the TyG index presents moderate power as a surrogate marker for recognizing HOMA-IR index in healthy subjects [4]. The TyG index has high sensitivity and specificity compared with the euglycemic hyperinsulinemic clamp test for recognizing IR [5]. The superiority of the TyG index in identifying IR was also reported in many other studies [6,7,8]. In the current study, TyG is more efficient than TG/HDL-C for identifying IR, supporting that both lipotoxicity and glucotoxicity have crucial roles in IR modulation.

To our knowledge, this is the first study to compare lipid, adipokines, and lipid and adipokine ratios, visceral adiposity indicators and TyG related parameters as surrogate markers of IR. TyG-BMI is a clinically useful surrogate in identification of IR, as it combines TG, FPG, and adiposity, and all the parameters are well validated for their roles in recognizing IR [30]. TyG-BMI positively correlated with HOMA-IR, and the ROC curve analysis suggested that TyG-BMI had the largest AUC (0.801), thus demonstrating its superior performance in recognizing IR than other variables. The availability of a simple and economical tool, such as TyG-BMI, is favorable for identifying high-risk individuals, thus expanding the benefits of screening. The ROC curve analysis showed that both TyG-BMI and LAR were effective in discriminating IR in the study population, revealing a similar capacity to discriminate this correlation. However, TyG-BMI, a simple and economical IR surrogate marker based on a single routine fasting blood test and BMI measurement, could have more favorable clinical values as an early indicator of IR risk.

Although the clinical appeal for the use of a measure of visceral fat is undeniable, results from studies that have attempted to compare different measurements of general and regional adiposity (BMI *vs*. WC) have not been consistent [32,33,34]. BMI is simple to measure and is commonly adopted as a useful indicator of general obesity and other metabolic abnormalities. Height and weight, used to calculate BMI, can be reliably measured, even though the BMI cannot distinguish body fat from fat-free mass. In our investigation, TyG-BMI performed more efficiently than TyG-WC did in identifying IR, possibly because WC, although easy to obtain, is an inaccurate measure of abdominal obesity [35]. Abdominal obesity includes subcutaneous and visceral adipose tissues. Visceral adipose tissues are crucial in identifying IR. WC alone cannot be used to differentiate between subcutaneous and visceral adipose tissues; therefore, WC cannot fully represent visceral adipose tissues. Imaging techniques, such as MRI and CT, can more accurately measure fat distribution and quantify adiposity; however, they are not readily accessible, economic, or usable in epidemiological studies. Another explanation is that WC is highly correlated with cardiometabolic risks; however, it is subject to inter-observer variability [33]. Unreliable measurement of the exposure variable can attenuate the observed association of exposure with the disease of interest, thereby reducing the power of the variable to detect a true association [36]. Possible measurement errors may arise from inadequate training and measurement difficulties. Inter-observer reliabilities for circumferences observed may range from 86% to 99% for WC [36]. In another study, the intra- and inter-observer variabilities of WC were higher than those of BMI [37]. In addition, WC differs depending on the precise site at which they are measured [38]. Reliable measurements of body fat and fat distribution are vital in epidemiological surveys. Anthropometric measurement error cannot be avoided but can be minimized by closely monitoring all aspects of data collection.

Our study has the following limitations. First, because of its cross-sectional design, the associations are not prospective and causality cannot be inferred. Further longitudinal study is necessary to confirm if the TyG-BMI index may predict future occurrence of IR. Second, because the study sample primarily includes Taiwanese individuals, the results cannot be generalized to other ethnicities; given the variability of TG levels according to ethnicity, further research is required to evaluate TyG-BMI in different populations.

## Conclusion

A simple and inexpensive method to measure IR for routine screening, prospective study, risk assessment, and therapeutic monitoring is essential. TyG-BMI comprehensively identifies and measures obesity and metabolic abnormalities in individuals. The simplicity of TyG-BMI calculation and low-cost biochemical measurements warrant further investigation of its role as a surrogate marker for IR to improve the identification of subjects with a high cardiometabolic risk and facilitate the prevention of chronic diseases associated with IR.
